# Dietary diversity and food intake of urban preschool children in North‐Western Sri Lanka

**DOI:** 10.1111/mcn.13006

**Published:** 2020-04-30

**Authors:** Fathima Sirasa, Lana Mitchell, Neil Harris

**Affiliations:** ^1^ Public Health, School of Medicine Griffith University Gold Coast QLD Australia; ^2^ School of Allied Health Sciences Griffith University Gold Coast QLD Australia; ^3^ Department of Applied Nutrition Wayamba University of Sri Lanka Makandura Sri Lanka; ^4^ Menzies Health Institute Queensland Australia

**Keywords:** child, diet quality, eating, feeding behaviour, serving size, Sri Lanka, urban population

## Abstract

Childhood malnutrition, associated with poor diet, is a clear public health threat in Sri Lanka, with high rates of under‐nutrition and micronutrient deficiencies coupled with the growing risk of overweight/obesity in urban locations. This study explored the dietary diversity and food intake of urban living Sri Lankan preschool children. A cross‐sectional analysis of the baseline data from a cohort study was conducted with parents/caregivers of children aged 2–6 years, from 21 preschool centres in Kurunegala District, Sri Lanka. Demographic and socio‐economic factors, dietary diversity score (DDS) (*n* = 597) and food intake (*n* = 458) (using a food frequency questionnaire) were assessed. Children had a mean DDS of 4.56 ± 0·85 out of 9, with most (91.1%) in the medium DDS category (DDS of 3.1–6.0), consuming rice as most common food. Lentils were consumed more than any meat or alternative food groups at all DDS levels. Child DDS differs with parent/caregiver age and ethnicity. Mean daily intakes of fruit (1.02) and vegetables (0.84) servings align with approximately half of national recommendations, with less than 20% of children meeting daily recommendations. More than one‐third consumed sugary snacks and confectionaries daily and 1 in 10 had them twice a day. Around 40% reported watching television while eating the evening meal. Despite the majority having reasonable DDSs (medium category), findings highlighted inadequate intakes of fruits and vegetables, excessive intakes of sugary snacks and unhealthy dietary and social behaviours, suggests the need for population‐based interventions to promote healthier dietary habits.

**Key messages**

- Diversity of children's diet is within the medium category (DDS of 3.1–6.0/9) for most (91.1%) and lentils were consumed more than any meat or alternative food groups at all DDS.
- Significantly higher child DDS was reported in children having parents/caregivers aged ≥60 years and/or belonging to Muslim ethnicity.
- Daily fruits and vegetables consumption was inadequate for more than 80% of children and 10% had sugary snacks and confectionaries twice a day.
- The unhealthy dietary and social behaviour of television watching while eating the evening meal was reported in 40% of children.
- Requirement for population‐based interventions to promote healthier dietary habits.

## 1 INTRODUCTION

Sri Lanka is a middle income country, experiencing rapid nutrition transition in response to urbanization and the globalized food system (Weerasekara, Withanachchi, Ginigaddara, & Ploeger, 2018). Traditional Sri Lankan diets rich in cereals and fibre, are being replaced with more western diets rich in sugar, fat, animal‐source products, and processed foods (Weerasekara et al., 2018). During this nutrition transition, both under and over‐nutrition and micronutrient deficiencies are reported in young children as diet‐related public health threats. In 2016, the prevalence of stunting, wasting and underweight in Sri Lankan children under five years of age were 17.3%, 15.1% and 20.5% respectively (Department of Census and Statistics & Ministry of Health, Nutrition and Indigenous Medicine, 2017). Further, the number of overweight and obese children has increased by 43% from 2006 to 2016 with the prevalence increasing from 0.8% to 1.5% in urban areas of Sri Lanka during this period (Department of Census and Statistics & Ministry of Health, Nutrition and Indigenous Medicine, 2017; Department of Census and Statistics & Ministry of Healthcare and Nutrition, 2009). Micronutrient deficiencies were also prevalent in children under five years of age, including iron (33.6%), calcium (47.6%) and vitamin A (29.3%) (Jayatissa, Gunathilaka, & Fernando, 2014; Rajapaksa, Arambepola, Gunawardena, Rosa, & Opatha, 2011). These nutritional outcomes are associated with poor dietary habits of children (Semba et al., 2010; Smith & Haddad, 2015; Yu et al., 2016).

A healthy diet is central for optimal nutritional status, health and well‐being of an individual. Dietary diversity is a key element of dietary quality, and nutrient adequacy (Kennedy, 2009). As reported in the 2012 National Nutrition and Micronutrient survey of Sri Lanka, the mean dietary diversity score (DDS) for children aged 24 to 59 months was 5.4 out of a possible 7 (Perkins, Jayatissa, & Subramanian, 2018). This indicates that, on average, the diets of Sri Lankan preschool children are of a moderate to high diversity. However, given the prevailing rates of child malnutrition, coupled with rapid industrial development, an updated assessment of DDS is important to understand the diversity of children's diet to inform effective strategies to promote healthy diets in Sri Lankan children. In general, DDS assessment does not take into account serving/portion sizes of intake. Therefore, determining the food intake servings along with the dietary diversity is necessary to clearly understand the dietary patterns.

Many countries have developed their own food or nutrient‐based dietary guidelines for different age categories and genders as the basis to promote healthy diets in individuals. Compared to the ‘Food based dietary guidelines for Sri Lankans’ (Ministry of Health, 2011), on average, Sri Lankan adults consume considerably lower servings of fruits, vegetables and dairy and greater servings of starch and added sugar (Jayawardena, Byrne, Soares, Katulanda, & Hills, 2013). To date, the food intake patterns of Sri Lankan children, especially of preschool age, have not been explored.

While undernutrition and micronutrient deficiencies are more widespread in rural areas, due to dietary changes in recent years, children in urban areas are at growing risk of over‐nutrition along with undernutrition and micronutrient deficiencies (Department of Census and Statistics & Ministry of Health, Nutrition and Indigenous Medicine, 2017; Marasinghe, Chackrewarthy, Abeysena, & Rajindrajith, 2015). Deficiencies in zinc (67%) and vitamin A (38%) have been shown to be prevalent in urban preschool children living in western Sri Lanka (Marasinghe et al., 2015). The dual burden of malnutrition is at least in part related to food choices and intake of children under age five (Haddad, Cameron, & Barnett, 2015). Since food intake patterns developed in childhood tend to track into adulthood, it is vital to promote healthy eating habits in the early years. Understanding dietary diversity of preschool children and their food intake patterns can assist to provide targeted guidance to foster healthy eating habits through appropriate interventions. Therefore, the present study was conducted with the aim of assessing dietary diversity and food intake of urban living preschool children in Sri Lanka.

## 2 METHODS

### 2.1 Study design and study population

A cross‐sectional analysis was conducted on the baseline data of a cohort study of preschool children living in urban North‐Western Sri Lanka between December 2017 and June 2018. The cohort study aimed to promote healthy eating, food knowledge and preferences in preschool children. Ethical approval was obtained from Griffith University Human Research Ethics Committee (GU Ref No: 2017/660 & 2017/812) and Wayamba University of Sri Lanka Ethics Review Committee (ERC No: 201810HI01). [Correction added on xx May 2020 after its first online publication. The ethical number obtained from Griffith University Human Research Ethics Committee has been added in this version.]

The sample was drawn from Kurunegala District (an urban area), North‐Western province of Sri Lanka, using a multistage convenience sampling strategy. Twenty‐five preschool centres (government and privately owned) were randomly selected using random‐number generation from a list of preschools registered with the Kurunegala District Secretariat, the local government authority. The centres were approached to participate and twenty‐one of them agreed. Parents or caregivers of the children aged 2–6 years at the consenting preschool centres (including 642 preschool children) were invited to participate with written informed consent obtained before data collection commenced.

### 2.2 Data collection

#### 2.2.1 Demographic and socio‐economics

Demographic and socio‐economic information of the parent/caregiver (age, sex, education level, occupation and family income) and the child (age and sex) were collected from parents/caregivers of the preschool children using a self‐administered questionnaire.

#### 2.2.2 Dietary diversity score (DDS)

DDSs were calculated for each child using the nine food groups of: rice, lentils, green leafy vegetables (green salads and ‘Mallum’), yellow/orange fruits, eggs, fish (including seafoods), chicken, meat other than chicken and milk (including all dairy based food products and excluding breast milk). The nine food groups selected were based on a DDS assessment conducted in Bangladesh for children aged less than five years old (Rah et al., 2010). Although the World Health Organization (WHO) has established guidelines to assess dietary diversity with respect to Infant and Young Child Feeding (IYCF) practices for children, their guidelines are specific for children aged 6–23 months (World Health Organization, 2010). Since our study sample is in the age group of 24–72 months, we adopted the method described in the study by Rah et al. (2010), which was conducted for the same age group in an Asian country (Bangladesh) consuming rice as staple food (similar to Sri Lanka). Parents/caregivers were asked to record the number of days, each of the above food groups were consumed in the previous week to determine the DDS (Rah et al., 2010). The DDS range a child could obtain was therefore 0–63, with the average daily DDS for each child calculated by dividing by seven.

#### 2.2.3 Food intake assessment (in servings and frequencies)

A short food frequency questionnaire (FFQ) was used to assess the food intake of preschool children. The original FFQ developed and validated by Flood et al. (2014), that included seventeen items, was adapted by replacing similar food items available in Sri Lanka. The questions were designed to assess usual intake, and included daily servings of fruit and vegetables, usual frequency of eating red meat, processed meats, high fat potatoes (hot chips, French fries or fried potatoes), salty snacks, take‐away food, snack foods (biscuits, cakes, doughnuts) and confectionery, cups of milk, fruit juice, sugary drinks (soft drinks, cordials, sports drinks, diet soft drinks or diet cokes) and water, frequency of eating the evening meal in front of the television and frequency of eating breakfast. An additional five questions about the preschool child's daily servings of rice (main staple in Sri Lanka), red meat, chicken and other meat, fish and egg were included in this questionnaire after pilot testing of the questionnaire and considering the ‘Food based dietary guidelines’ developed for preschool children in Sri Lanka (Ministry of Health, 2011) (Appendix A).

Standard household measures were used to obtain food serving sizes such as cup, coconut shell spoon, different piece sizes (for fruits and animal source food), can and bottle (for beverages). These measures were explained clearly using food serving size photographs and parents/caregivers were provided with clear instructions with examples on how to record the food and beverages their child usually eats. Serving size equivalents used for different food groups were mentioned in the FFQ (Appendix A).

Data were collected by a five‐member research team (final year food and nutrition undergraduate students) supervised by a nutritionist. Team members were trained on assessment before the data collection and to minimize missing data, completed questionnaires were cross‐checked prior to participants leaving the study location. When there was a discrepancy in the dietary data, participants were contacted via mobile telephone with information clarified using alternative cross‐checking questions. Illiterate participants were assisted by the research team to complete the questionnaire. Data were entered by research team members and entries were double‐checked by the supervising nutritionist.

### 2.3 Statistical analyses

Statistical analyses were conducted using SPSS version 25.0 (SPSS Inc., 2017). Descriptive data are presented as mean and standard deviation (SD). Due to incompatibility of the targeted age group of children in our study with the target group specified in WHO guidelines, DDS assessment conducted by Rah et al. (2010) for children <5 years was used. Since the Rah et al. (2010) study did not define DDS cut off values and Sri Lanka does not have national cut‐off values for low, medium and high DDSs, tertiles were derived from the 9 food groups and used to classify the children into low (≤3.0), medium (3.1–6.0) and high (6.1–9.0) dietary diversity based on methods used in similar studies (Ajani, 2010; Habte et al., 2015; Ogechi & Chilezie, 2017).

Percentages of consumption of different food groups according to DDS categories were calculated. Average DDS values were rounded up to the nearest whole number to get individual DDS values. Independent *t* test, ANOVA (or the Mann–Whitney *U* test and Kruskal–Wallis test if not normally distributed) and post‐hoc procedures were used to compare mean values. Average daily intake of serving sizes for selected food and beverage groups were calculated. The serving size for meat or alternatives was computed by summing the serving sizes of red meat, chicken and other meat, fish and egg. Legumes/lentils were not included in the meat or alternatives group since they were categorized in the vegetables group in this study (for DDS, lentils were separately grouped). Participants who responded as either ‘don't know/can't say’ or ‘refused to answer’ were removed from the specific serving size calculations. Mean daily consumption of each food or food group was analysed according to gender and two age categories (24–47 months and 48–72 months). Independent *t* tests were used to determine the differences between the mean values of serving sizes between genders and age groups. A *p‐*value <0.05 was considered as significant in all analyses.

## 3 RESULTS

### 3.1 Participant characteristics

The demographic and socio‐economic characteristics of the study sample are shown in Table 1. The sample included similar proportion of children from both genders, with the majority in the age category of 48–72 months (87.7%). The majority of participating parents/caregivers were females (88.3%), aged less than 39 years (78.8%), belonging to Sinhalese ethnicity (77.1%) and housewives (65.0%). Around 33% of the parents/caregivers completed the Ordinary Level educational qualification (Completed Grade 11) and more than half reported a monthly household income of 20,000LKR‐45,000LKR (USD 111.2–248.0).

**TABLE 1:** Demographic and socio‐economic characteristics and dietary diversity score of the children aged 24–72 months of age included in the study (*n* = 597)

			Average DDS			
Subject characteristics	*n*	%	Mean	SD	95% CI	*p‐*value^c^
**Child (Total)**	597	100	4.56	0.85	[4.49, 4.63]	
Age (months)^a^						
24–47	63	12.3	4.70	0.98	[4.45, 4.94]	0.133
48–72	448	87.7	4.50	0.84	[4.42, 4.58]	
Gender^a^						
Male	277	51.0	4.53	0.84	[4.43, 4.63]	0.728
Female	266	49.0	4.54	0.87	[4.44, 4.65]	
**Parent/caregiver**						
Age (years)^a^						
< 29	136	22.8	4.61^A^	0.83	[4.47, 4.76]	**0.002**
30–39	334	56.0	4.52^A^	0.84	[4.43, 4.61]	
40–49	92	15.4	4.46^A^	0.82	[4.29, 4.63]	
50–59	15	2.5	4.89	0.96	[4.36, 5.42]	
≥ 60	19	3.2	5.22^B^	1.01	[4.73, 5.71]	
Gender						
Male	70	11.7	4.53	0.90	[4.32, 4.74]	0.737
Female	527	88.3	4.56	0.85	[4.49, 4.64]	
Ethnicity						
Sinhalese	460	77.1	4.51^A^	0.88	[4.43, 4.59]	**0.018**
Muslim	106	17.8	4.73^B^	0.75	[4.59, 4.88]	
Tamil	25	4.2	4.74	0.78	[4.42, 5.07]	
Malay	6	1.0	4.22	0.50	[3.69, 4.74]	
Level of Education^a^						
No education	4	0.7	4.20	0.83	[2.88, 5.52]	0.585
Up to Grade 5	12	2.0	4.55	0.75	[4.08, 5.02]	
Grade 6–10	61	10.3	4.62	0.83	[4.41, 4.84]	
Completed Grade 11	193	32.7	4.53	0.88	[4.40, 4.65]	
Grade 12–13	81	13.7	4.73	0.73	[4.57, 4.89]	
Completed Grade 13	137	23.2	4.55	0.85	[4.40, 4.69]	
Degree and above	102	17.3	4.53	0.91	[4.35, 4.71]	
Occupation^a^						
House wife	385	65.0	4.57	0.81	[4.48, 4.65]	0.234
Professional/technical/managerial	112	18.9	4.55	0.86	[4.39, 4.72]	
Self employed	29	4.9	4.53	0.84	[4.21, 4.85]	
Clerical	19	3.2	4.76	0.94	[4.31, 5.21]	
Sales and services	18	3.0	4.73	1.03	[4.22, 5.24]	
Other^b^	26	4.4	4.20	1.07	[3.77, 4.64]	
Not employed	3	0.5	5.27	1.11	[2.52, 8.01]	
Household income (LKR/month)^a^						
< 20,000	88	15.5	4.59	0.92	[4.39, 4.78]	0.124
20,000 ‐ < 30,000	151	26.5	4.46	0.77	[4.33, 4.58]	
30,000 ‐ < 45,000	154	27.1	4.62	0.86	[4.48, 4.76]	
45,000 ‐ <60,000	100	17.6	4.45	0.84	[4.29, 4.62]	
60,000 ‐ < 80,000	38	6.7	4.70	0.83	[4.42, 4.97]	
80,000 or more	38	6.7	4.80	1.03	[4.46, 5.14]	

SD, standard deviation; CI, Confidence Interval

a Missing values for child age (*n* = 86), child gender (*n* = 54), caregivers' age (*n* = 1), caregivers' level of education (*n* = 7), caregivers' occupation (*n* = 5) and caregivers' family income (*n* = 28).

b Other includes skilled manual, unskilled manual, agriculture, armed forces and elementary workers.

c Mean DDS comparisons were made using Independent *t* test, ANOVA (or the Mann–Whitney U test and Kruskal–Wallis test if not normally distributed) and post‐hoc procedures.

Different uppercase superscript letters indicate significant differences (*p* < 0.05) in child DDS between categories of demographic socio‐economic characteristics as determined by post‐hoc procedure.

### 3.2 Dietary diversity

Baseline results for DDS were available from 597 children (Table 1). The mean DDS of the preschool children was 4.56 (SD 0.85) out of a possible 9 (Table 1). DDS did not significantly differ by child's age or gender. Age of the parent/caregiver and ethnicity showed significant differences in child's DDS. Significantly higher child DDS was reported in older parents/caregivers aged ≥60 years (5.22 ± 1.01) compared to young parents/caregivers aged <29 years (4.61 ± 0.83, *p* = 0.028), 30–39 years (4.52 ± 0.84, *p* = 0.004) and 40–49 years (4.46 ± 0.82, *p* = 0.003). No difference was observed between the parents/caregivers aged ≥60 years and 50–59 years (*p* = 0.794). Children belonging to Muslim ethnicity (4.73 ± 0.75, *p* = 0.047) showed significantly higher DDS compared to Sinhalese ethnic children (majority of the population) (4.51 ± 0.88). There were no significant differences between other ethnic groups.

Of the study population, DDS values range from 2 to 7 out of 9 (2.4 to 6.7). The majority of children (91.1%, *n* = 544) fell within the medium DDS category, while 4.4% and 4.5% were in the low and high DDS categories respectively (Table 2). The percentage consumption of each food group increased as the DDS value or category increased (Table 2). Rice, the staple food of Sri Lankans, was consumed by 87.9% of children in the low DDS category (≤3.0). In this category, 44.5% consumed milk and milk products and one‐third consumed lentils, however less than one‐third consumed each of the other food categories. More than half of participating children consumed milk and milk products with a DDS of 3 or more, whereas it increased to a DDS of 5 for lentils, green leafy vegetables and yellow/orange fruits. The most common food groups in the medium DDS category are: rice, milk and milk products, yellow/orange fruits, green leafy vegetables and lentils. Those in the high and low DDS category are slightly more likely to consume lentils than yellow/orange fruits or green leafy vegetables. For all DDS categories, a greater proportion of children consumed lentils compared with any meat or alternative food groups (egg/fish/chicken/meat other than chicken). Egg and fish were consumed by more than half of participants with a DDS value of 6 and those in the high DDS category.

**TABLE 2:** Percentage of children with DDS and percentage consumption of different food groups by DDS for children aged 24–72 months (*n* = 597)

	Individual DDS^a^ (average DDS range)						DDS categories (average DDS range)		
	2 (1.5–2.4)	3 (2.5–3.4)	4 (3.5–4.4)	5 (4.5–5.4)	6 (5.5–6.4)	7 (6.5–7.4)	Low DDS (≤3.0)	Medium DDS (3.1–6.0)	High DDS (6.1–9.0)
Number of children	2	66	212	233	77	7	26	544	27
Percentage of children with DDS	0.3	11.1	35.5	39.0	12.9	1.2	4.4	91.1	4.5

	Percentage consumption of food groups at individual DDS^a^						Percentage consumption of food groups at DDS categories		
Food groups	2 (1.5–2.4)	3 (2.5–3.4)	4 (3.5–4.4)	5 (4.5–5.4)	6 (5.5–6.4)	7 (6.5–7.4)	Low DDS (≤3.0)	Medium DDS (3.1–6.0)	High DDS (6.1–9.0)
Rice	71.4	91.6	97.5	98.1	99.1	100.0	87.9	97.5	100.0
Lentils	21.4	35.1	45.7	59.8	75.5	81.6	33.5	53.7	84.1
Green leafy vegetables	14.3	34.6	44.7	58.7	79.2	87.8	23.6	54.1	78.8
Yellow/orange fruits	50.0	30.5	46.6	61.7	73.5	81.6	30.2	54.5	80.4
Egg	35.7	29.7	41.4	49.5	61.0	79.6	25.3	46.0	72.0
Fish	21.4	21.9	29.4	41.8	56.4	65.3	19.8	36.6	67.2
Chicken	7.1	13.0	17.5	24.6	35.3	42.9	13.2	21.8	41.8
Meat other than chicken	0.0	2.4	4.3	9.1	11.7	22.4	2.7	7.1	11.6
Milk and milk products	21.4	55.4	79.2	89.0	98.5	100.0	44.5	84.0	99.5

a Individual DDS values were obtained by rounding up the average DDS values to the nearest whole number. Individual DDS values of 1, 8 and 9 were not available in the present sample.

### 3.3 Food intake (and dietary behaviour)

Food intake information was available from 458 children. The mean daily intake for each of 13 FFQ food types/groups, according to two age categories and genders are shown in Table 3. On average, participating children consumed 2.42 servings of rice, 0.84 servings of vegetables, 1.02 servings of fruits, 1.56 servings of meat or alternatives and 1.14 cups of milk per day. Whole milk was consumed by majority (92.3%) of children and almost all used milk powder (96.4%) to prepare milk or milk added beverage (data not shown). Boys consumed significantly higher daily serves of chicken and other meat compared to girls (0.45 vs 0.32, *p* < 0.05), as well as milk (1.24 vs 1.04, *p* < 0.05). Children of younger age category (24–47 months) ate significantly more serves of fish compared to the older age category (48–72 months) (0.74 vs 0.50, *p* < 0.05), and also eggs (0.64 vs 0.50, *p* < 0.05). Mean daily water intake (cups) was significantly higher in the older age category children than the younger category (3.71 vs 3.12, *p* < 0.05).

**TABLE 3:** Average daily intake of food groups (serving/day) by preschool children (*n* = 458)

		Total children		Age categories				Gender			
				24–47 months		48–72 months		Males		Females	
FFQ food group (servings/day)	*n*	Mean	SD	Mean	SD	Mean	SD	Mean	SD	Mean	SD
Rice	458	2.42	1.29	2.19	1.25	2.44	1.29	2.50	1.31	2.34	1.26
Vegetable	448	0.84	0.71	1.02	0.79	0.82	0.70	0.83	0.67	0.85	0.74
Fruits	446	1.02	0.79	1.11	1.01	1.02	0.77	1.05	0.86	1.00	0.72
Meat or alternatives	359	1.56	0.98	1.76	1.73	1.55	0.89	1.46	0.94	1.66	1.00
Red meat	387	0.15	0.34	0.08	0.15	0.16	0.35	0.17	0.38	0.13	0.30
Chicken and other meat	422	0.39	0.46	0.46	0.68	0.38	0.43	**0.45** *	0.52	**0.32** *	0.38
Fish	455	0.52	0.45	**0.74** *	0.88	**0.50** *	0.38	0.55	0.44	0.49	0.46
Egg	457	0.52	0.31	**0.64** *	0.38	**0.50** *	0.30	0.51	0.31	0.52	0.31
Milk^a^	457	1.14	0.83	1.17	0.71	1.14	0.84	**1.24** *	0.85	**1.04** *	0.80
Fruit juice^a^	446	0.35	0.44	0.33	0.28	0.36	0.45	0.37	0.45	0.33	0.42
Soft drink^a^	436	0.14	0.24	0.09	0.13	0.14	0.25	0.14	0.23	0.14	0.25
Water^a^	446	3.65	1.71	**3.12** *	1.50	**3.71** *	1.72	3.56	1.65	3.75	1.77

SD, standard deviation

a Cups of intake was included instead of servings.

* Significantly different within age categories or gender at *p* < 0.05.

Table 4 and 5 presents food servings and frequency for each of the food groups consumed per day, week or month respectively. Rice consumption was reported as minimum of 3 servings/week and maximum of 7 servings/day. More than 80% did not reach the minimum recommendation for fruits and vegetables (2 servings/day) (Ministry of Health, 2011). Meat or alternatives consumption exceeded the recommendation in 5.6% of children (Ministry of Health, 2011). Daily recommended intake of water (750 mL to 1 L) (Ministry of Health, 2011) was not met by 37% of children. Soft drink was not consumed by 40% of the children, whereas around one‐third of children consumed one or more cups at least once weekly and 2.5% consumed daily. No children in the present study reported consuming diet soft drinks or diet cokes.

**TABLE 4:** Percentage of children according to the food servings consumed from different food groups (*n* = 458)

FFQ food group		Percentage of children											
			Per month	Per week						Per day			
	*n*	Not consumed	≥1	≥1	≥2	≥3	≥4	≥5	≥6	≥1	≥2	≥3	≥4
**Servings of intake**													
Rice	458	1.1	‐	‐	‐	98.9	97.2	97.2	96.3	96.1	71.6	42.8	14.0
Vegetable	448	2.5	97.5	96.2	90.6	78.6	46.9	44.6	42.9	42.2	13.6^a^	3.1^a^	0.4^a^
Fruits	446	1.6	‐	98.4	95.1	87.4	76.2	65.2	57.0	56.1	17.3^a^	6.3^a^	1.3^a^
Meat or alternative	359	0.8	‐	‐	99.2	98.6	95.3	91.1	86.4	79.4^a^	22.3^a^	5.6^a^	1.9^a^
Red meat	387	71.8	‐	27.6	21.7	16.3	11.6	7.0	5.4	3.4	1.3	0.3	0.0
Chicken and other meat	422	13.0	87.0	85.5	61.6	38.2	23.2	13.5	11.1	7.6	2.4	1.2	0.2
Fish	455	4.2	95.8	95.6	83.7	59.3	37.8	22.4	17.1	13.0	3.1	0.4	0.2
Egg	457	1.5	98.5	98.2	89.9	67.6	43.5	25.4	15.3	12.7	0.9	0.2	0.0
Milk^b^	457	15.1	84.9	84.2	83.2	79.4	74.2	72.9	71.3	70.9	30.9	5.0	0.4
Fruit juice^b^	446	18.4	81.6	71.1	55.2	35.9	22.0	13.7	10.3	10.1	2.5	0.7	0.2
Soft drink^b^	436	40.1	59.9	35.3	21.6	11.0	6.4	4.1	2.8	2.5	0.5	0.0	0.0
Water^b^	446	0.9	‐	‐	99.1	98.9	98.7	98.7	98.7	98.7	94.8	63.0^a^	45.7^a^

a Meets national recommended intake: vegetables = ≥2 servings /day; fruits = ≥2 servings /day; meat or alternatives = 1–2 servings/day; water = 750‐1,000 mL (3–4 cups) /day.

b Cups of intake was included instead of servings.

**TABLE 5:** Percentage of children according to the frequency of consumption from different food groups (*n* = 458)

FFQ food group		Percentage of children											
			Per month	Per week						Per day			
	*n*	Not consumed	≥1	≥1	≥2	≥3	≥4	≥5	≥6	≥1	≥2	≥3	≥4
**Frequency of intake**													
Red meat	411	74.5	25.5	23.6	19.7	14.4	7.8	4.1	3.6	2.9	1.2	‐	‐
Processed meat products	431	65.2	34.8	23.9	14.4	7.7	5.3	3.2	2.3	2.1	1.4	0.9	0.9
High fat potatoes	438	61.4	38.6	24.4	15.3	8.2	5.5	3.7	2.5	2.3	0.7	0.7	0.2
Salty snacks	458	31.0	69.0	60.0	48.7	33.0	20.7	14.2	10.7	9.2	2.6	0.7	‐
Take‐away foods	436	69.3	30.7	12.4	4.6	2.3	1.6	0.9	0.5	0.5	0.2	0.2	‐
Snacks (mostly biscuits)	458	16.3	83.7	78.3	72.0	61.3	50.9	42.4	37.6	36.5	12.8	3.3	1.5
Confectionery	458	9.0	91.0	88.2	84.7	75.8	61.6	50.4	41.9	40.0	14.2	4.8	1.3
Breakfast	451	4.0	96.0	95.1	94.7	91.8	89.6	87.8	84.9	84.7	‐	‐	‐
Dinner while watching television	452	41.2	‐	58.6	57.5	54.2	48.0	44.7	40.9	39.4	‐	‐	‐

One‐quarter of children consumed red meat more than once a month, with more than half of these consuming it at least 3 times per week (Table 5). Around 25% of children sampled consumed processed meat products and high fat potatoes (hot chips, French fries or fried potatoes) at least once per week. Over a third of participating parents/caregivers reported their child having consumed confectionery (40.0%) and snacks (mostly biscuits) (36.5%) once or more daily and salty snacks were consumed daily by one in ten children. Only 11% of children missed their regular breakfast and 4% did not ever consume breakfast. More than half of the study population had their dinner while watching television and nearly 40% followed this routine daily.

## 4 DISCUSSION

In this study, a mean DDS of 4.56 (out of 9) was recorded for 2–6 years olds, which is comparatively lower than the mean DDS value (5.4 out of 7) reported in 2012 National Nutrition and Micronutrient survey among under 5 year olds in urban Sri Lanka (Perkins et al., 2018). The current study found that 4.4% of children scored low DDS, which is slightly lower than the proportion of under five year old Sri Lankan children (6%) who did not meet minimum dietary diversity requirements as reported in the 2012 National Nutrition and Micronutrient survey (Perkins et al., 2018). In comparing the findings of the present study with Perkins et al. (2018), it is important to note the different usage of food groups (type and number) and different scoring systems used. Although the Sri Lanka National Nutrition and Micronutrient survey was conducted in 2012, the results were only published in 2018 (Perkins et al., 2018). This prevented the usage of the DDS assessment method described in the Sri Lanka National Nutrition and Micronutrient survey in our study, which was conceptualized, designed and data collected before the publication of national survey results including methodology.

Using nine food groups to calculate the DDS has been reported in studies of young children in other middle‐income countries (Kennedy, Pedro, Seghieri, Nantel, & Brouwer, 2007; Steyn, Nel, Nantel, Kennedy, & Labadarios, 2006). According to the Kennedy et al. (2007) study, Filipino children aged 24–71 months showed mean DDS of 4.91, which is similar to our study findings (mean DDS = 4.56), whereas in South African children aged 1–8 years, comparatively lower mean DDS (3.6) was reported (Steyn et al., 2006).

DDS values of children with older caregivers (≥60 years) were significantly greater than that of children having younger caregivers aged <49 years. The older caregivers (≥60 years) were grandparents of these children (*n* = 19). In Sri Lanka, the role of grandparents has evolved toward that of a primary caregiver (not full‐time) due to increased female (mother) labour force participation (Department of Census and Statistics, 2017; Premaratne, 2011). Grandparents often use food as an emotional tool to express love and care (Jingxiong et al., 2007) and a US study reported that grandparents offer their portion of fruit and vegetables to their grandchildren (Speirs, Braun, Zoumenou, Anderson, & Finkbeiner, 2009). Parents prefer grandparents to take up the child care responsibilities of their children rather than day care centres, due to the comparatively higher cost and uncertain quality of day care centres (Premaratne, 2011). While the shift in labour force participation is often viewed as a negative for families, research suggests spending more time with grandparents positively influences the diet of grandchildren. A recent study conducted by Farrow (2014) reported that when grandparents spend more time in caring for their grandchildren, they provide a healthier food environment and greater modelling of healthy food habits to grandchildren than do parents. The culture of child‐care through long day care centres is not well established in Sri Lanka. However, about 76% of 3–5 year‐old children attend some form of learning program (preschool or entry grade in basic education) (Dundar et al., 2017), normally conducted for 4–5 hours in the mornings until about midday. Therefore, the child is still in the care of the parent/caregiver the majority of the day. Additionally, meals consumed by children at preschool centres are brought from home.

Children belonging to the Muslim ethnic group living in urban areas scored greater mean DDSs than children from the Sinhalese ethnic group (majority of the population). Although the same food is available for consumption by all ethnic groups, religious norms among these ethnic groups shape their cultural food beliefs and practices. Sinhalese ethnic groups do not eat red meat as a religious norm prevents the killing of animals for consumption. Townsend et al. (2015) also reported reduced intake of meat and meat products in Sri Lankan secondary school children (most belong to Sinhalese ethnic group). Thus, ethnic group membership could contribute to the lower DDS reported in children belonging to Sinhalese ethnic group. Further, in our study, ‘meat other than chicken’ (especially red meat) was reported as the least consumed food group (Table 2) which is likely also due to the cost of red meat.

In our study, the inclusion of a variety of food groups was observed even with the lower DDS values. Lentils were more commonly consumed than yellow/orange fruits or green leafy vegetables or any meat or alternative food groups (egg/fish/chicken/meat other than chicken). This could be due to the regular incorporation of red lentils into daily meals of Sri Lankans (Anoma, Collins, & McNeil, 2014; Thavarajah et al., 2011). Red lentils are popular among Sri Lankans because of the cooking convenience and relatively low cost compared to meat or alternatives as a protein source (Anoma et al., 2014). Sri Lanka imports red lentils, with the government of Sri Lanka recognizing lentils as one of the essential commodities (Parliament of the Democratic Socialist Republic of Sri Lanka, 2007).

To our knowledge, this study is the first to provide data on food intake patterns in serving sizes and frequencies among urban living preschool children in Sri Lanka. On average, children consumed approximately half of their daily recommended intake of fruits (1.02) and vegetables (0.84), where the national recommendation is ≥2 servings/per day. This finding was in line with the results of a Malaysian study conducted to assess the fruit and vegetable intake patterns among children aged 1–6 years (Chong, Lee, Ng, Khouw, & Poh, 2017). On average, Malaysian children consumed 0.91 and 1.07 servings of fruits and vegetables respectively. In the Malaysian study, less than one‐fifth of children achieved the daily recommended servings for fruits and vegetables, which is similar to the results of current study in Sri Lankan context, where recommendations were met by 17.3% for fruits and 13.6% for vegetables. Similar results were reported in Canadian preschool children, in which 16.6% met the recommended servings (≥ 5servings) for fruit and vegetables together (Dubois, Farmer, Girard, Burnier, & Porcherie, 2011).

The national recommendation for cereals and starchy foods (including cereal based preparations and tubers) for pre‐school age children is 3–4 servings/day (Ministry of Health, 2011). Rice intake was the primary food assessed in this category in our study, since it is the staple cereal of Sri Lankans. Tubers/yams were categorized in the vegetables group according to our FFQ. Although this study did not assess the intake of other cereals and cereal based preparations, around 43% of children met the minimum recommendation for cereals and starchy foods based only on rice intake, and nearly 14% exceeded the recommended level (4 servings/day) (Ministry of Health, 2011). Children's mean daily intake of meat or alternatives (1.56 servings) was aligned with the national recommendation of 1–2 servings/day (Ministry of Health, 2011). While the national recommendation for meat or alternatives includes legumes/lentils, our study did not include legumes/lentils in this food group for computation of the servings as they were categorized in the vegetables group due to the nature of questions. Around 80% of children achieved the lower range of the recommendation (1 serving/day) for meat or alternatives, which indicates that these children eat at least one type of food daily from this food group (red meat/chicken and other meat/fish/egg). Our findings showed slightly higher percentages compared to a previous study, which revealed that 64% of Ethiopian children consumed animal source foods (which includes dairy in addition to meat, poultry, fish and egg) (on previous day of the survey) when they lived in urban areas (Herrador et al., 2015). This is advantageous, as the intake of the meat or alternatives food group predicts nutrient adequacy and supports better nutritional status in children (Ruel, 2003).

Younger children (24–47 months) consumed more fish and egg than the older preschool children (48–72 months). This finding may be explained by parental awareness of child nutrition acquired through nutrition education programs via family health services conducted throughout Sri Lanka. Parents gather information on appropriate complementary feeding practices for their young child mainly via the Child Health Development Record (62%) followed by the family health services midwives (28.7%) (Seram & Punchihewa, 2017). Fish and egg are high‐quality protein sources and rich in essential fatty acids (FA) (*n‐3* FA in fish) (*n‐3* FA and *n‐6* FA in egg) which are important for the cognitive and visual development in young children (Eilander, Hundscheid, Osendarp, Transler, & Zock, 2007; Iannotti, Lutter, Bunn, & Stewart, 2014). They may have also chosen fish as a softer meat type and therefore, easier for young children to chew and swallow.

Our study found that male children consumed more ‘chicken and other meat’ and ‘milk’ than female children. A similar finding was observed for milk consumption in Indian children less than five years old (Fledderjohann et al., 2014). Our finding evidences a slight gender disparity whereby male children are given preferential treatment including intake of growth promoting nutrient rich foods (protein from ‘chicken and other meat’ and calcium from ‘milk’) compared to female children. However, there were no gender differences found for the intake of the other nine food groups and this may be due to the young age group of our study population. Previous studies conducted in Nepal (Gittelsohn, 1991) and India (Aurino, 2017) reported that gender‐based disparities in food distribution or nutrient intake were not observed in young children, but emerge at the onset of adolescence.

The proportion of children that consumed soft drink daily (2.5%) in our study is extremely low compared to US children aged 24–48 months (46%) (Kay, Welker, Jacquier, & Story, 2018) and Indonesian children aged 24–35 months (60%) (Green et al., 2019). Indonesian mothers reported the child's preference and demand for sugar‐sweetened beverages (SSB) as the prominent factor for providing the child with SSB (Green et al., 2019). More than one‐third of children had at least daily consumption of snacks (mostly biscuits, 36.5%) and confectionaries (40%). These findings add support to previous research documenting increased consumption of sugary snack foods (including biscuits, confectionary and soft drinks) in Asia, especially in low‐and‐middle income countries (Huffman, Piwoz, Vosti, & Dewey, 2014). Huffman et al. (2014) assessed sugary snack foods consumption among 6–23‐month‐old children in 18 countries in Asia and Africa. They found that a considerable proportion of Asian children (42–75%) in their age of 12–24 months, consumed sugary snack foods (on the day preceding the interview).

The national recommendation for foods containing sugar is to limit the intake and only consume in small amounts (Ministry of Health, 2011). Although this is not quantifiable as servings or frequencies, the statement implies sugary snack foods are not recommended for daily intake. Accordingly, more than one‐third of our study population exceeded the recommendation for sugary snack foods, which is an unhealthy sign for a growing young generation. The main reasons behind this high incidence of sugary snack food intake by preschool children could be the wide availability of these foods even in small grocery shops all over Sri Lanka (Townsend et al., 2015), innate preference for sweet taste in childhood (Ventura & Mennella, 2011) and strong advertising of sugary snack foods and beverages by multinational companies through television (Ferando, Achala Abeykoon, & Pushpika Kumari Ganegoda, 2015; Prathapan, Wijewardena, & Low, 2016). A recent study conducted a content analysis of food and beverages advertisements targeting children and adults on television in Sri Lanka found that 78% of the advertisements were child focused; confectionaries were one of the most common types of food that were advertised targeting children; and among the child‐focused advertisements, nearly 90% were categorized as unhealthy (Prathapan et al., 2016). Further, such snack and beverages are low priced compared to prices of comparable healthy food choices (Townsend et al., 2015).

Comparatively, a low proportion of the present sample consumed salty snacks (9.2%) and high fat potatoes (hot chips, French fries or fried potatoes) (2.3%) daily when compared with US preschool children (26.9%, 18.5% respectively) (Fox, Condon, Briefel, Reidy, & Deming, 2010). This could be due to increased snacking pattern (Piernas & Popkin, 2010) and reporting consumption of French fries as most common vegetables (Fox et al., 2010) in the USA, which were not observed in Sri Lanka. Having dinner (evening meal) daily while watching television was observed in around 40% (*n* = 178) of the studied children. Studies have shown that there is an increased risk of overweight and obesity in preschool children with higher intakes of snack foods that are sugary (DeBoer, Scharf, & Demmer, 2013; Gubbels, Kremers, Goldbohm, Stafleu, & Thijs, 2012; Linardakis, Sarri, Pateraki, Sbokos, & Kafatos, 2008; Millar et al., 2014; Yu et al., 2016), and/or salty/high fat (Gubbels et al., 2012; Millar et al., 2014) as well as watching television during meals and snacks (Dubois, Farmer, Girard, & Peterson, 2008). Therefore, focusing on these unhealthy dietary patterns in future interventions is imperative.

On a positive note, the majority (84.7%) of children were reported as daily breakfast eaters, similar to findings reported in Canadian (Dubois, Girard, Kent, Farmer, & Tatone‐Tokuda, 2009) and Malaysian (Norimah et al., 2014) preschool children. Interestingly, this figure is substantially greater than the 37% that was reported in Sri Lankan children aged 4–12 years in 2008 (Senanayake & Parakramadasa, 2008). This positive finding could be due to the practice of suggested menus to bring in for meal times by most preschool centres. In Sri Lanka, preschool centres operate from early morning to midday. So, children bring their meal (mostly breakfast) to the centre and have it just before the mid‐morning break at around 10 o'clock.

The present study has several limitations. While a multistage sampling strategy was used, recruitment was from one district (Kurunegala) of two in the North‐Western province of Sri Lanka. This decision was made due to time and resource constraints. Therefore, findings must be generalized with caution to all urban children in North western province of Sri Lanka. To diversify the sample drawn, preschool centres were selected randomly from the study location (Kurunegala district). All children attending the selected centres were invited to participate, and those who provided parent/caregiver consent and completed the questionnaires by parent/caregiver were considered as the study population. This sampling strategy could lead to selection bias, as those parents/caregivers more interested in health and nutrition may have been more willing to participate. Additionally, the DDS was constructed using a simple count of food groups over the previous 7‐day reference period. Information on serving sizes or number of serves was not obtained. Therefore, this DDS could only reflect the diversity of the diet rather the quality of diet. The FFQ used in this study was not tested for validity or reliability with Sri Lankan young children. However, it included questions on food groups rather than individual food items and a few food groups were added to the FFQ after pilot testing to minimize measurement error. Food intake assessment was based on parental reporting with the acknowledged possibility of measurement bias due to under and over‐reporting linked with social desirability.

The present study has several strengths. The DDS used a reference period of the previous 7‐days and scoring was based on a weighted system approach (Ruel, 2003). The weights reflect the number of days the foods or food groups were consumed over a 7‐day reference period. So, this method provides a better view of the diversity of the diet than a DDS calculated using a single day 24‐hr recall. We collected extensive information on dietary patterns of young children including serving sizes and frequencies. Further, this is the first study to describe the dietary habits of preschool children in urban Sri Lanka. The key information on prevailing dietary issues in this urban preschool cohort would be the initiation point to health care professionals and policy makers of Sri Lanka to extend this investigation at the national level. Further studies at the national level or to compare different locations (urban vs rural and/estate) would be helpful to understand the dietary issues of preschool children more clearly and aid in development or modification of related policies and effective interventions to promote healthy diets in Sri Lankan children.

## 5 CONCLUSIONS

The findings revealed even though the majority of children in our study population consumed a diet within the medium category of diversity (DDS of 3.1–6.0), most of them do not consume an adequate amount of fruits and vegetables and more than one‐third consume excessive sugary snacks. A considerable proportion of children follow the unhealthy dietary and social behaviour of television watching while eating the evening meal. This suggests that there is a need for population‐based interventions to promote healthy diets and higher dietary diversity among preschool children and their families in this urban cohort.

## CONFLICTS OF INTEREST

The authors declare that they have no conflicts of interest.

## CONTRIBUTIONS

FS conceptualized and designed the study, collected data, analysed the data and drafted the manuscript; LM and NH involved in study design, supervised the study and critically reviewed the manuscript. All authors contributed substantially to revisions of the manuscript, read and approved the final manuscript.
